# Challenges and Successes in Raising a Child With Type 1 Diabetes and Autism Spectrum Disorder: Mixed Methods Study

**DOI:** 10.2196/17184

**Published:** 2020-06-03

**Authors:** Tamara K Oser, Sean M Oser, Jessica A Parascando, Lee Ann Grisolano, Kanthi Bangalore Krishna, Daniel E Hale, Michelle Litchman, Shideh Majidi, Paul Haidet

**Affiliations:** 1 Department of Family Medicine University of Colorado School of Medicine Aurora, CO United States; 2 Department of Family and Community Medicine Penn State College of Medicine Hershey, PA United States; 3 Department of Psychiatry Penn State College of Medicine Herhsey, PA United States; 4 Division of Pediatric Endocrinology Department of Pediatrics Penn State of College of Medicine Hershey, PA United States; 5 College of Nursing University of Utah Salt Lake City, UT United States; 6 Barbara Davis Center University of Colorado School of Medicine Aurora, CO United States; 7 Departments of Medicine, Public Health Sciences, and Humanities Penn State College of Medicine Hershey, PA United States

**Keywords:** type 1 diabetes, autism spectrum disorder, child, blogs, social media, qualitative research

## Abstract

**Background:**

Self-management of type 1 diabetes (T1D) requires numerous decisions and actions by people with T1D and their caregivers and poses many daily challenges. For those with T1D and a developmental disorder such as autism spectrum disorder (ASD), more complex challenges arise, though these remain largely unstudied.

**Objective:**

This study aimed to better understand the barriers and facilitators of raising a child with T1D and ASD. Secondary analysis of web-based content (phase 1) and telephone interviews (phase 2) were conducted to further expand the existing knowledge on the challenges and successes faced by these families.

**Methods:**

Phase 1 involved a qualitative analysis of publicly available online forums and blog posts by caregivers of children with both T1D and ASD. Themes from phase 1 were used to create an interview guide for further in-depth exploration via interviews. In phase 2, caregivers of children with both T1D and ASD were recruited from Penn State Health endocrinology clinics and through the web from social media posts to T1D-focused groups and sites. Interested respondents were directed to a secure web-based eligibility assessment. Information related to T1D and ASD diagnosis, contact information, and demographics were collected. On the basis of survey responses, participants were selected for a follow-up telephone interview and were asked to complete the adaptive behavior assessment system, third edition parent form to assess autism severity and upload a copy of their child’s most recent hemoglobin A_1c_ (HbA_1c_) result. Interviews were transcribed, imported into NVivo qualitative data management software, and analyzed to determine common themes related to barriers and facilitators of raising a child with both ASD and T1D.

**Results:**

For phase 1, 398 forum posts and blog posts between 2009 and 2016 were analyzed. Common themes related to a lack of understanding by the separate ASD and T1D caregiver communities, advice on coping techniques, rules and routines, and descriptions of the health care experience. For phase 2, 12 eligible respondents were interviewed. For interviewees, the average age of the child at diagnosis with T1D and ASD was 7.92 years and 5.55 years, respectively. Average self-reported and documented HbA_1c_ levels for children with T1D and ASD were 8.6% (70 mmol/mol) and 8.7% (72 mmol/mol), respectively. Common themes from the interviews related to increased emotional burden, frustration surrounding the amount of information they are expected to learn, and challenges in the school setting.

**Conclusions:**

Caregivers of children with both T1D and ASD face unique challenges, distinct from those faced by caregivers of individuals who have either disorder alone. Understanding these challenges may help health care providers in caring for this unique population. Referral to the diabetes online community may be a potential resource to supplement the care received by the medical community.

## Introduction

### Background

Individuals with type 1 diabetes (T1D) spend over 8000 hours per year self-managing diabetes outside of the medical setting [1]. Optimal management includes numerous daily behaviors and decisions such as checking blood glucose levels, counting carbohydrates, calculating doses of and administering insulin, and changing dietary habits. Following this regimen is challenging for anyone, but for people with developmental disorders, such as autism spectrum disorder (ASD), this complex care can be even more demanding. Individuals with ASD can have impairments in social communication and interaction as well as repetitive or restrictive behaviors and preferences [2]. Behaviors and activities that individuals with ASD perceive as unimportant or do not understand, such as self-care, health and safety awareness, and activities of daily living, can be very difficult for them to manage independently, which can make the complexity of self-managing diabetes even more challenging. In addition, over 30% of people with ASD also have an intellectual disability (ID), which can add additional challenges in the self-management of T1D [3].

T1D prevalence is increasing, affecting 1.25 million people in the United States in 2014, and 5 million people are expected to be affected by 2050 [4-6]. Up to 1 in 59 children in the United States have been identified with ASD [7], and the reported prevalence of ASD is also increasing [3,7]. There is little research regarding the prevalence of individuals with both ASD and T1D in the United States [3,6-8], although a recent study found the prevalence of ASD in the pediatric T1D population in 1 clinic to be 1.16% (95% CI 0.96-1.26) [9]. An additional study reported that among 10,032 participants from the T1D Exchange Clinic Network, 159 (1.58%) had T1D and ASD [10]. These studies suggest that there is no increased prevalence of ASD in children with diabetes. However, research has documented that people with ID alone may be more likely to develop T1D as a result of having genetic conditions such as Down syndrome [11,12]. In addition, 31% of children with ASD also have an ID [3]. Although ASD alone may not lead to increased diabetic ketoacidosis, severe hypoglycemia, or worsening glycemic control [10], T1D patients with both ASD and comorbid ID are more likely to have very poor glycemic control, higher emergency room utilization, and lower rates of achieving national standards of diabetes management, including lower rates of lipid screening and retinopathy screening [11-13]. With this comes greater risk for negative outcomes, and indeed diabetes-related morbidity and mortality have been identified as a particular concern for people with ID [14,15]. As people with ASD and ID live longer, it has become even more important that they achieve better long-term disease management, including glycemic control and control of other modifiable risk factors, to decrease the occurrence of potentially preventable complications as they age. The interplay between ASD with or without ID and impacts on clinical, behavioral, and psychosocial outcomes related to T1D continue to be areas where little has been explored. Many factors may be involved in such interplay and outcomes, including access to social support and social networks [16], which may be valuable resources in raising a child with both T1D and autism.

Although there have been great advances over the last decades in health care delivered to children with chronic conditions, not all children have benefited equally [17]. Although there is a well-developed evidence base regarding diabetes care and education for populations without ASD or ID, very little research has been done to better understand optimal ways to deliver education and behavioral interventions and to align clinical care with the needs of families raising a child with both T1D and ASD, with or without an ID.

### Objectives

This study sought to better understand self-management challenges and successes experienced by families with experience in raising a child with both T1D and ASD to lay the groundwork for future studies. This will enable the exploration of interventions to improve diabetes outcomes and reduce health disparities in this special population and other unique populations of children living with diabetes and other chronic medical conditions. To the best of our knowledge, this is the first study that utilizes a qualitative component to allow further exploration of the lived experience of raising a child with both T1D and ASD. We also sought to gather some exploratory information on glycemic control related to the degree of adaptive function in light of the coexisting ASD, hypothesizing that adaptive impairment may be associated with hemoglobin A_1c_ (HbA_1c_).

## Methods

### Design and Ethics

This study involved an innovative approach consisting of 2 phases and included the use of social media as well as traditional interviews to capitalize on the strengths of each. In the first phase, secondary data analysis of web-based content was conducted to understand the challenges and successes families raising a child with T1D and ASD experience, similar to our other studies using web-based content to evaluate barriers and facilitators of living with or raising a child with T1D [18,19]. The themes generated from this study were used to create an interview guide that was used in phase 2 to interview caregivers of children with varying degrees of glycemic control and ASD severity to provide richer exploration of the lived experience in raising a child with both T1D and ASD. This 2-step approach allowed further exploration of initial themes identified from the secondary data analysis of web-based content, with the advantage of being able to ask follow-up questions.

This study was approved by the Penn State College of Medicine institutional review board through expedited review and was considered to be *no greater than minimal risk.* No procedures or participant interactions took place before receiving this approval.

### Phase 1

#### Recruitment

No individuals were actively recruited for the first phase of the study, as it consisted of a secondary data analysis of publicly available data. Content from a publicly available forum and 2 blogs were identified through an internet search using the search terms *type 1 diabetes and autism* and *diabetes and autism*, which would capture both *autism* and *autism spectrum disorder*.

#### Analysis

Qualitative methods are appropriate for generating new knowledge and hypotheses, especially when little is known about the area of investigation [20]. This type of analysis allows the emergence of themes from the data when there are no existing hypotheses to test, such as in this understudied population of children living with both T1D and ASD.

A total of 2 experienced qualitative researchers (TO and SO) undertook data analysis. Content from online forums and blogs were imported into NVivo version 12 qualitative software (QSR International Pty Ltd) post by post. After reviewing blogs and forum posts and noting initial impressions, a codebook was developed and revised through ongoing discussions among the study team. To establish Cohen κ (a measure of interrater reliability in qualitative coding) [21], the primary coders (TO and SO) each coded 20% of the dataset (κ=0.996). With the established interrater reliability, the remaining posts and blog comments in the full dataset were coded. The research team employed an exploratory inductive thematic approach to construct emergent themes [22]. Themes from the analysis of web-based content were used to create an interview guide for use in the second phase of the study.

### Phase 2

#### Recruitment

For the second phase of the study, participants were recruited on the web, through physician referral, and with flyers posted in the waiting room of an academic pediatric endocrinology practice. Participants were considered eligible if they were a caregiver (parent, grandparent, adult sibling, or guardian) to a child aged 5-18 years with both T1D and ASD; able to read, write, and communicate effectively in English; and had telephone and internet access. A summary explanation of the research was provided, including implied consent upon reading the summary of explanation and agreeing to participate.

#### Instrument

The eligibility survey was hosted in Research Electronic Data Capture (REDCap), a secure electronic data collection system [23]. The survey was tested for technological function before distribution, and questions were answered on 1 page. REDCap automatically captures the date of survey completion and completeness of the survey and assigns each participant a unique study code number. Participants were unable to go back in the survey and change their answers. The survey could only be completed once per Internet Protocol address. Participants reported demographics and information related to their child’s conditions and diabetes provider.

#### Interview

Survey participants were also asked if they would consent to a subsequent 30-min telephone interview. The interview guide was developed based on the thematic qualitative analysis of web-based content in phase 1. This included themes that seemed fully or nearly fully contextualized by the web-based data as well as themes that may not have been as fully developed, as the phase 2 interviews were used to assess the potential completeness of the phase 1 themes as well as to elicit deeper and more nuanced exploration of the phase 1 themes. To do this, a random sample of interview participants from online recruitment sources was selected for interviews based on consent and responses to the survey questions. As certain demographic groups (eg, clinic patients) were underrepresented in this sample, we also purposively chose to interview the few clinic patients who completed the survey (n=2). Such a targeted, purposive approach to adding participants is standard in qualitative research, as it is 1 of the distinct features that increases efficiency and yield, thereby minimizing overrepresentation and unneeded effort from participants in groups adequately represented [24]. This strategy enabled an interview sample large enough to reach saturation in the interviews, as is also typical of such qualitative research [24].

Telephone interviews were scheduled based on the earliest convenience of the participant. The interviews lasted approximately 30 min and were conducted by 3 authors (PH, SO, and TO). Interviews were audio recorded on a hand-held device and then transferred to a secure web-based storage location with access limited to the study team and an approved Health Insurance Portability and Accountability Act (HIPAA)-compliant transcriptionist.

After scheduling the interview, participants received a link to the adaptive behavior assessment system, third edition (ABAS-3) parent form [25], also hosted in REDCap, and were instructed to complete it either before or after the telephone interview. The ABAS-3 parent form assesses the functional skills of children and adolescents who display various types of limitations, disabilities, or disorders, much like those subjectively displayed by children with ASD and ID. The items included *assess everyday activities required, eg, to function adaptively, respond to environmental demands, care for oneself, and interact with others*. The results of the ABAS-3 identify, among other things, strengths and limitations as well as the need for services and support [25,26]. The general adaptive composite (GAC) scores were derived from all of the adaptive skills areas in the assessment and ranged from 40 to 120. Higher scores indicate a greater level of daily functioning, whereas lower scores suggest impairments in any of a variety of adaptive areas that are assessed by the ABAS-3. The further the GAC score deviates downward from what is considered typical of an individual’s age, the greater the impairment in function. For example, a GAC score of 65 would suggest a greater level of impairment in adaptive behavior than a GAC score of 95.

Participants were also asked to upload a copy of the child’s most recent HbA_1c_ result (within the past 4 months) into the REDCap survey. After completing all procedures, participants were compensated for their time and effort with a gift card.

#### Analysis

In all, 12 respondents were interviewed. Interviews were transcribed using HIPAA-compliant Penn State transcription services. Interview transcripts were imported into NVivo 12 (QSR International) for analysis and analyzed as detailed above through an exploratory inductive thematic qualitative approach (Cohen κ after 20%=0.942). Attempts were made to include caregivers of children with varying degrees of glycemic control and varying degrees of function related to their ASD.

Descriptive statistics were calculated for ABAS-3 parent form data and for HbA_1c_ results were also calculated by subgroups based on HbA_1c_ being <7.5% (<58 mmol/mol) or ≥7.5% (≥58 mmol/mol) according to then-current American Diabetes Association glycemic target recommendations [27].

## Results

### Phase 1

There were 398 posts between 2009 and 2016 from a publicly available forum for caregivers raising a child with both T1D and ASD (117 unique members) and 25 individual blog posts from 2 blogs authored by a caregiver to a child with T1D and ASD. In all, 4 themes emerged from phase 1 (Table 1).

**Table 1:** Phase 1 themes and representative quotes from analysis of online forums and blogs.

Themes	Representative web-based quotes
Parents of children with T1D^a^ and autism yearned for support and did not feel understood by the autism community or the T1D community; social media provided much needed peer support from families that understood the unique experience of raising a child with both conditions.	“I feel left out of the T1D groups because Aspergers brings a whole new set of concerns. But the Aspie groups are completely afraid of T1D.” “Like you said—the autism community is not equipped for our extra needs and the diabetes community just doesn’t get the autism side of it.” “I found this group less than a week ago, and WEPT tears of joy that there are other parents out there who know what this is like.” “I am so thrilled to have found this group! I knew there were other families like ours but I didn’t know how/where to access them. I am in tears right now because I feel so overwhelmed knowing that there are other people who understand the everyday trials (and victories!) both autism and diabetes bring to your life 24/7.”
Numerous coping techniques were discussed, including focusing on T1D management first, as glucose levels could affect behavior.	“What has worked is focusing on diabetes management first. When his blood sugars are high he is so irritable and easily set off.” “We chose to focus on T1D first because I do believe that has a huge effect on his behavior.”
Descriptions of the health care experience, including the helpfulness of multidisciplinary teams with endocrinologists and autism specialists jointly developing treatment plans.	“But the Endocrinologists I've encountered don't seem to see it that way. Ours acts like autism and diabetes are as related as a sprained ankle + appendicitis. To them, diabetes is about numbers and autism doesn't affect those. (Ha!)” “Psychiatrists have only a passing familiarity with insulin dependence from an hour or two during med school, and they are mostly only interested in how it intersects with the meds they prescribe. So again, you as the advocate have to navigate the separate systems, trying to educate whoever is willing to listen along the way.” “My suggestion would be that the Endo consult with your regular doctor. A treatment and medical management plan should be put into place between the two offices. You may also ask for nutritionist, OT and behavior consults to help your child learn more about his diabetes and how his anger issues play into his blood glucose levels.”
Sensory issues precluding the use of technology such as insulin pumps and the utility of tubeless pumps in overcoming some of these challenges.	“My son is on shots, BTW. We tried a pump for a brief time but he wouldn't tolerate it. He pulled it off up to three times a day.” “We went with the tubeless pump for the reasons that have come up...we were concerned about our son messing with the controls and we also knew that for a kid with sensory issues, the external tubing would be a real problem.” “There was NO WAY my son could have dealt with tubing, but he’s not having problems with the tubeless pump, we've been very pleased.” “There is just no way my sensory son would have tolerated tubing so I am glad we pursued the tubeless pump option.” “The one thing that helped with sensory issues for things attached [insulin pump, CGM] was I started putting bandaids on for everything-- every shot, bump, bruise, etc. After a few years he hardly noticed them...and as long as the tubing is tucked in he won’t use that as something to self-stimulate with.”

^a^T1D: type 1 diabetes.

### Phase 2

In phase 2, 12 participants were selected for a telephone interview from among 29 respondents who completed the eligibility survey. Information related to participant demographics and study variables such as HbA_1c_ and ABAS-3 parent form scores were obtained (Table 2). On average, both interview participants and the broader group of survey respondents were similar in current age, age of child’s diagnosis with T1D, age of child’s diagnosis with ASD, and self-reported HbA_1c_. For both interview participants and survey respondents, the majority reported that they were white, not Hispanic or Latino, lived in a suburban setting, and visited their child’s provider every 3-6 months. The majority of both groups reported using continuous glucose monitoring (CGM). The majority of survey respondents reported using an insulin pump, whereas interview participants were evenly split.

In the study sample, 8 individuals had the most recent HbA_1c_ >7.5%, with an average of 9.7% (SD 1.89), or 82.5 mmol/mol (SD 20.5); 4 individuals had the most recent HbA_1c_ <7.5%, averaging 6.9% (SD 0.29), or 52.8 mmol/mol (SD 2.9). There was no statistically significant difference in GAC scores between those with HbA_1c_ >7.5% (mean 68.6, SD 14.22) and those with HbA_1c_ <7.5% (mean 76.3, SD 11.73; t_10_=0.92; *P*=.38).

Overall, 3 themes emerged from an inductive thematic analysis of content obtained from interviews during phase 2 (Table 3).

**Table 2:** Phase 2 participant characteristics.

Variable		Interview participants (n=12)	Survey respondents (not interviewed; n=17)
Child’s current age (years), mean (SD)		12.3 (3.1)	12.6 (3.7)
Child’s age at diagnosis with T1D^a^ (years), mean (SD)		7.9 (2.8)	7.3 (3.4)
Child’s age at diagnosis with ASD^b^ (years), mean (SD)		5.6 (3.5)	5.6 (3.8)
Self-reported HbA_1c_^c^, % (mmol/mol)		8.6 (70)	7.7 (61)
Laboratory confirmed HbA_1c_, % (mmol/mol)		8.7 (72)	N/A^d^
**Country of residence, n (%)**			
	United States	12 (100)	17 (100)
**Gender, n (%)**			
	Male	7 (58)	10 (59)
	Female	5 (42)	7 (41)
**Race, n (%)**			
	White	11 (92)	16 (94)
	Black	0 (0)	1 (6)
	More than 1 race	1 (8)	0 (0)
**Ethnicity, n (%)**			
	Hispanic or Latino	1 (8)	0 (0)
	Not Hispanic or Latino	11 (92)	16 (94)
	Unknown/not reported	0 (0)	1 (6)
**Setting, n (%)**			
	Suburban	10 (83)	10 (59)
	Urban	2 (17)	4 (23)
	Rural	0 (0)	3 (18)
**CGM^e^ use, n (%)**			
	Yes	8 (67)	13 (77)
	No	4 (33)	4 (23)
**Insulin pump use, n (%)**			
	Yes	6 (50)	14 (82)
	No	6 (50)	3 (18)
Tubeless pump use among subset of pump users, n (%)		5 (83)	7 (50)
**Distance to provider, n (%)**			
	0-10 miles	3 (25)	4 (23)
	11-20 miles	2 (17)	3 (18)
	21-50 miles	7 (58)	6 (35)
	>50 miles	0 (0)	4 (23)
**Frequency of provider visits (usual care is every 3 months), n (%)**			
	<3 months	1 (8)	0 (0)
	3-6 months	11 (927)	16 (94)
	>6 months	0 (0)	1 (6)
ABAS-3^f^ GAC^g^ standard score, mean (95% CI)		71.2 (67.9-74.4)	N/A

^a^T1D: type 1 diabetes.

^b^ASD: autism spectrum disorder.

^c^HbA_1c_: hemoglobin A_1c._

^d^N/A: not applicable.

^e^CGM: continuous glucose monitoring.

^f^ABAS-3: adaptive behavior assessment system, third edition.

^g^GAC: general adaptive composite.

**Table 3:** Phase 2 themes and representative quotes from analysis of interviews.

Theme	Representative interview quotes
Caregivers of children with T1D^a^ and autism face emotional burdens that may be more than additive compared with raising a child with only T1D or autism, and they describe the constant monitoring and work required to care for their children.	“Autism doesn’t go away because of a diabetes diagnosis.” “How are we going to do this forever?” “I am a nervous wreck every day.” “She doesn’t show any signs of being low until she is on the brink of losing consciousness and then all of a sudden she is almost passing out.” “There is a different kind of fear when it comes to diabetes than with autism.” “Things are very overwhelming---I cried myself to sleep a couple of nights last week.” “We are working harder than any other parent in the diabetes clinic and no matter what we do or how much monitoring, or how much insulin we give, it’s like nothing seems to help.” “We try so hard and it seems like it’s not doing anything.” “He cannot tell me if his blood sugar is low or high so I always have to be right there, constantly monitoring.”
Caregivers of children with T1D and autism express frustration surrounding the amount of T1D information they are expected to learn and the manner in which it is presented.	“Sometimes people should understand that the best learning is from somebody teaching you and not from reading it.” One caregiver describes a nurse educator who told her “you should know how to do this already. Just change the insulin. You should know how to do that already,” and her concern “that’s a really dangerous statement to make to a parent who’s new, who you never taught to change those doses.” “They showed me how to put it [the CGM] in and sent me on my merry way, which was the hardest thing ever to learn. I had to then put it in on my own and it took me three tries and I ruined two sensors. I felt so bad because I didn’t know how to do it.” Another caregiver described how a health care provider “handed me the Pink Panther [reference book regarding type 1 diabetes] and thought I had read it all.” “I feel like especially with the hurdles we deal with, a lot more education from the onset would be very helpful.” Caregivers offered solutions such as “make step by step videos like the ‘how to’ videos seen on YouTube” and offering “extra appointments and longer appointments.”
Caregivers describe numerous challenges surrounding their child’s experience in the school setting.	“I am literally on call even when she’s at school. Even though she has an aide and there’s a nurse, they will still call me to come help take care of her on a daily basis.” “They’ve dealt with her eloping from the classroom for years and they know she’s a good hider. They knew about the diabetes. I mean, I know they’re only human, but my goodness. If you lose a diabetic autistic child, that’s kind of a big deal.” “I stepped down from my position as a paralegal to take care of my child and I now work at my daughter’s school, just to make sure that she is ok.” Multiple caregivers described how they had to take “time off”, “reduce to part-time,” or even “take jobs at the school to be available to assist with [their] child’s care”. “Schools are not doing the best they can to help these kids. Sometimes they think the kids can do it on their own where you know they have more challenges and they need more help.” “No one can work together to get us what we need.”

^a^T1D: type 1 diabetes.

## Discussion

### Principal Findings

Caregivers raising a child with comorbid T1D and ASD face unique challenges that are not well understood by families with a child with only 1 of the individual conditions or by health care providers, as evident by the themes that emerged from phase 1. Themes were generally related to the combination of T1D and ASD and focused on coping techniques, advice on needles and technology, health care experience, and sensory issues related to technology (eg, insulin pumps). Themes from phase 2 are complementary. Although some could be interpreted as applying to nearly anyone performing T1D management (with or without coexisting ASD), many of the diabetes-specific challenges were influenced (and generally amplified) by the coexistence of ASD.

To the best of our knowledge, this is the first study with a large qualitative component to better understand the experience of raising a child with both T1D and ASD. The use of both social media and interviews as qualitative data sources in this special population provided a deeper understanding than using either type of data source alone, much like we found in another study using social media and interviews in a sample of adults with T1D [18].

Overall, our study results show that children who have greater impairment in function based on adaptive skills areas do not achieve target HbA_1c_ values. On the basis of the themes that emerged from our interviews (phase 2), this may be due to the additional barriers that come with the comorbidity, overwhelming amount of information to learn, and challenges in the school setting. More detailed comparisons around our exploratory aim were not conclusive due to the small sample size, not surprisingly.

Caregivers who shared the many challenges they face in raising a child with T1D and ASD might benefit from expanded social networks to provide social support, including emotional, instrumental, informational, and appraisal support. Both in-person support and referral to components of the online caregiver community specific to comorbid T1D and ASD could be valuable resources, as supportive ties may enhance well-being and health of the caregivers, which has been shown to lead to positive outcomes in children with T1D [28-34]. Social networks can provide much needed emotional support, exemplified by 1 participant who, upon finding such a web-based resource, “WEPT tears of joy that there are other parents out there who know what this is like.” Instrumental support might result if caregivers to a child with comorbid T1D and ASD find they are geographically able to provide assistance such as babysitting services, because finding caregivers for any child with T1D presents challenges, and these challenges may be significantly increased in a child with both T1D and ASD [19]. This study shows that caregivers identified numerous coping techniques, including focusing on T1D management first, establishing concrete rules and routines, advice on types of needles and technology, and suggestions for health care appointments. Prioritizing disease-specific tasks when diabetes coexists with other health conditions has been reported elsewhere [35]. Referral to social networks where this kind of informational support can be shared may be highly valuable. Appraisal support in the form of feedback may be especially useful to caregivers with a child newly diagnosed with either T1D or ASD in the setting of having previously been diagnosed with the other condition, as receiving feedback that the challenges they are facing are real and being handled to the best of the caregiver’s ability may help the caregiver in coping with the added new diagnosis.

**Figure 1:**
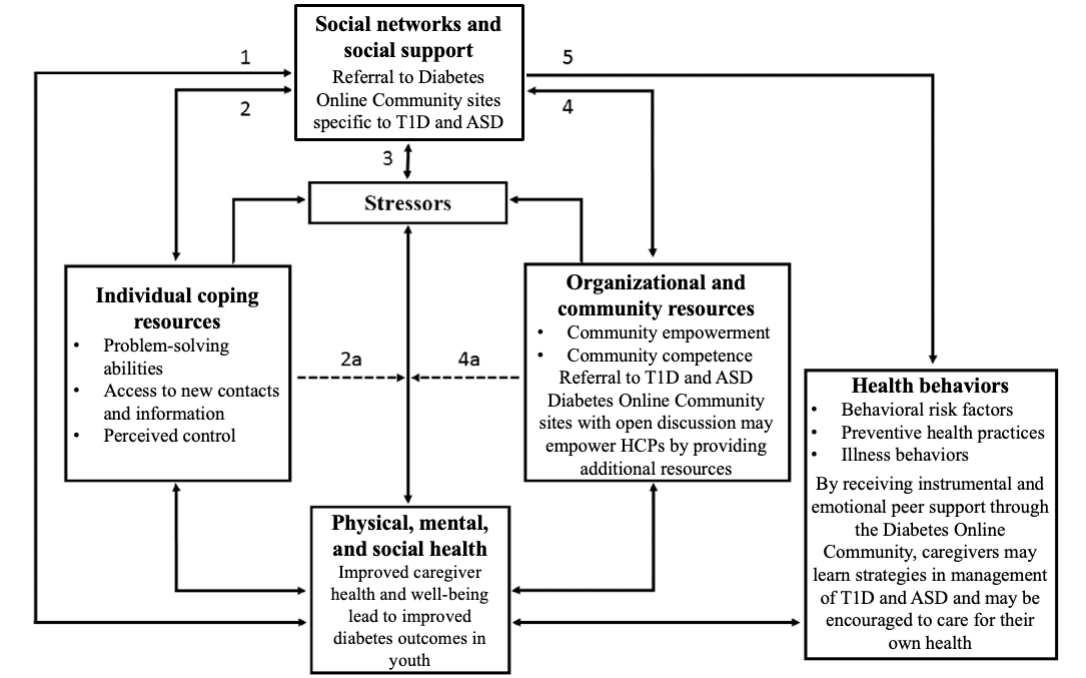
A modified conceptual model for the relationship of social networks and social support to health, specific to type 1 diabetes and autism spectrum disorder. ASD: autism spectrum disorder; HCP: health care practitioner; T1D: type 1 diabetes.

A modified conceptual model for the relationship of social networks and social support to health can be applied to this unique population (Figure 1, adapted from Glanz et al [16]). Pathway 1 shows that through the expansion of social networks, the supportive ties developed may enhance caregiver health and well-being, which is linked in multiple studies to improved diabetes outcomes in their youth. Pathway 2 represents the hypothesized effect of social networks and social support on individuals’ coping resources. By being able to access new contacts and information to help solve the unique challenges faced by families raising a child with T1D and ASD, there may be a sense or perception of increased control that may also improve the physical and mental health of caregivers. The community of health care providers caring for these children should consider referral to web-based caregiver sites specific for families raising children with both T1D and ASD. By providing resources to these families to strengthen their social networks, the community of health care providers caring for these children may also feel more empowered in caring for this challenging population, as shown in pathway 4. Pathways 2a and 4a demonstrate the *buffering effect*, whereby the negative effects of the stressful experience of raising a child with T1D and ASD described in this study may be diminished through an expanded social network and having health care providers who understand the potential role of peer support in coping. Pathway 3 illustrates how receiving information through an expanded social network may reduce the amount of stress experienced, which, in turn, can lead to improved mental and physical health in caregivers of children with T1D and ASD. Finally, pathway 5 describes the potential effects of social networks and support on health behaviors. By exchanging information and support, caregivers to a child with T1D and ASD may be more supported in health behaviors that improve the care of their child and themselves as well.

### Comparison With Previous Work

Our study demonstrated that the majority of survey respondents (8/12, 67%) and interview participants (13/17, 77%) had a child who used CGM technology. However, 82% (14/17) of the survey respondents reported their child using an insulin pump, compared with only 50% (6/12) of interview participants. Among those using insulin pumps, the majority use tubeless pumps (12/20, 60% overall, 5/6, 83% of interviewees and 7/14, 50% of survey respondents).

These findings complement the work of Stanek et al [9] and Bethin et al [10], who found that CGM use was the same among children with T1D and ASD as it was among those with T1D but without ASD, whereas insulin pump use was lower for those with both T1D and ASD. This qualitative work adds some explanatory context, as caregivers identified that sensory issues can preclude the successful use of insulin pumps and that successful solutions for some have included the use of tubeless pumps and desensitization techniques, such as the routine use of band-aids before attempting to use diabetes technology.

HbA_1c_ among study participants was not statistically correlated with the degree of function related to ASD, but it was limited by sample size. However, the trends of 2 comparisons (mean GAC scores of those achieving target HbA_1c_ vs those not achieving target HbA_1c_ and correlation of GAC scores with HbA_1c_) were both in the direction that one would expect: children who scored higher in skills of daily living had improved HbA_1c_ values (<7.5%). It is not surprising that this study did not achieve statistical significance, as it was not powered to do so and the sample size was chosen for the greater qualitative focus here. This also echoes comments by Bethin et al [10] and Stanek et al [9] that further work is needed to be able to better support these children and their families. From this study specifically, we propose that further research with larger sample sizes is needed to continue to explore the potential relationships between the degree of functioning related to ASD and glycemic control.

### Limitations

Limitations of this study include the lack of representation of caregivers from diverse ethnic backgrounds and rural areas. In addition, although attempts were made to recruit both from an endocrinology clinic and the diabetes online community (DOC), a minority of participants were recruited through the clinic. An additional limitation is that the search strategy included *autism* to capture ASDs but did not specifically include *Asperger* and may, therefore, have limited the scope of search returns. However, this would not fully exclude representation of Asperger’s-related content as it is an ASD, and it would also tend to skew the sample toward more severe levels of impairment, increasing the ability to identify challenges within the sample. This study aligns with previous work involving the DOC that demonstrates a larger web presence of white individuals living in suburban or urban areas, which is not likely representative of the greater population with comorbid T1D and ASD [36]. This may represent a health disparity whereby those in rural areas or from diverse ethnic backgrounds may not be aware of the DOC, may not have access, or may not feel welcome or included in that space.

### Conclusions

Raising a child with both ASD and T1D presents significant self-management challenges. However, caregivers of this population identify various coping techniques and strategies and find support in social media sites specific to this unique population. Further research is needed to develop new ways for the health care community to partner with this population.

## Abbreviations

ABAS-3: adaptive behavior assessment system, third edition
ASD: autism spectrum disorder
CGM: continuous glucose monitoring
DOC: diabetes online community
GAC: general adaptive composite
HbA_1c_: hemoglobin A_1c_
HIPAA: Health Insurance Portability and Accountability Act
ID: intellectual disability
REDCap: Research Electronic Data Capture
T1D: type 1 diabetes
